# Generation of iPSC lines from archived non-cryoprotected biobanked dura mater

**DOI:** 10.1186/2051-5960-2-4

**Published:** 2014-01-07

**Authors:** Andrew A Sproul, Lauren B Vensand, Carmen R Dusenberry, Samson Jacob, Jean Paul G Vonsattel, Daniel J Paull, Michael L Shelanski, John F Crary, Scott A Noggle

**Affiliations:** 1 The New York Stem Cell Foundation Research Institute, New York, NY 10032, USA; 2 Department of Pathology & Cell Biology and the Taub Institute for Research on Alzheimer’s Disease and the Aging Brain, Columbia University, New York, NY 10032, USA

## Abstract

**Background:**

Induced pluripotent stem cells (iPSCs) derived from patients with neurodegenerative disease generally lack neuropathological confirmation, the gold standard for disease classification and grading of severity. The use of tissue with a definitive neuropathological diagnosis would be an ideal source for iPSCs. The challenge to this approach is that the majority of biobanked brain tissue was not meant for growing live cells, and thus was not frozen in the presence of cryoprotectants such as DMSO.

**Results:**

We report the generation of iPSCs from frozen non-cryoprotected dural tissue stored at −80°C for up to 11 years. This autopsy cohort included subjects with Alzheimer’s disease and four other neurodegenerative diseases.

**Conclusions:**

Disease-specific iPSCs can be generated from readily available, archival biobanked tissue. This allows for rapid expansion of generating iPSCs with confirmed pathology as well as allowing access to rare patient variants that have been banked.

## Background

Human iPSC-derived neural cells are attractive models for Alzheimer’s disease and other neurodegenerative diseases because they can be used for cellular investigation of mechanisms and drug screening *in vitro*. While, to date, most human iPSC models have been derived from rare monogenetic familial forms of neurodegenerative disease, most patients have sporadic disease forms for which post-mortem neuropathological examination is essential for definitive diagnosis. In some cases, tissue from patients with similar symptoms may exhibit quite different pathology. For instance, vascular dementia or frontotemporal lobar degeneration, which can clinically present as AD, may not be correctly diagnosed until post mortem examination of the brain. If tissue from deceased patients that had undergone neuropathological evaluation could be used to generate iPSCs, knowledge of the definitive diagnosis as well as potential stratification of sporadic patients could guide the selection and subsequent use of the cell lines to be made. While generation of iPSCs from fresh autopsy tissue has recently been reported [1,2], brain bank networks, which contain tens of thousands of samples, could provide a much larger, and more immediate, source of tissue. As these biobanked tissues have not been specifically processed for the derivation of living cells, we have investigated whether it is possible to use them to isolate somatic cells and subsequently reprogram these into iPSCs. This would allow access to large numbers of neuropathologically defined cases, including patients with rare diseases whose frequency is low in clinical populations.

## Methods

### Cell culture

All media components were obtained from Life Technologies unless otherwise indicated. Dural/scalp outgrowths, established fibroblast lines, and mouse embryonic fibroblasts (MEFs, GlobalStem) were grown in fibroblast media, defined as the following: DMEM/10% FBS/Glutamax (2 mM)/2-Mercaptoethanol (0.1 mM)/Penicillin-streptomycin (100 U/mL-0.1 mg/mL). For initial plating of new dural and scalp samples, the tissue was first grown in biopsy media: DMEM/10% FBS/Glutamax (2 mM)/2-Mercaptoethanol (0.1 mM)/MEM non-essential amino acids (0.1 mM)/antibiotic-antimycotic (1×)/Nucleosides (1×, Millipore). Reprogrammed iPSCs were maintained on MEFs, in HUESM: KO-DMEM/20% KSR/Glutamax (2 mM)/2-Mercaptoethanol (0.1 mM)/bFGF (10 ng/ml)/Penicillin-streptomycin (100 U/mL-0.1 mg/mL). iPSCs were enzymatically passaged using TrypLE and replated in the presence of a Rho-kinase inhibitor (Y27632, Stemgent). Karyotyping and DNA fingerprinting of fibroblasts and iPSCs was performed by Cell Line Genetics (Madison, Wisconsin). Directed and undirected differentiations are described below.

### Generation of postmortem tissue outgrowths

De-identified donated postmortem brain tissue was obtained through the New York Brain Bank at Columbia University. Neuropathological examination was per standardized protocols [3,4]. For the pilot study, scalp and dural tissue from the same patient was frozen at the time of autopsy in the presence of 10% DMSO/45% FBS/45% fibroblast media. Subsequent experiments utilized standard banked material that was frozen via a liquid nitrogen vapor sandwich method [3,4]. Dural tissue was stored as rolled tissue (approximately 1 cm by 5 cm in 2 ml cryovials that have been stored at −80 degrees for 1–11 years. Samples were either thawed entirely for 1 min at 37°C for processing or quickly removed from the vial with forceps while still frozen and a small piece was cut off using a scalpel, to preserve unthawed tissue for future use. Samples were washed twice with PBS and DMEM, then cut into smaller pieces, approximately 2–3 mm by 2–3 mm. One drop of sterile silicon grease was placed in the center of each well of a 6-well cell culture plate and four or five pieces of the tissue were placed around each drop. A coverslip was placed on top of the silicon/samples and 2 ml of biopsy culture media were added to each well. Samples were left undisturbed for five days and then checked for fibroblast outgrowth. Media was switched to fibroblast culture medium and changed every other day. Outgrowth was monitored and fibroblasts were passaged with TrypLE to a new 6-well culture dish when the coverslip and/or plate became at least 50% confluent. Fibroblast cultures were expanded and passaged until sufficient numbers were generated for reprogramming and cryogenic preservation.

### Reprogramming using Sendai virus

Fibroblasts between passages 3 and 5 were plated into a 12-well cell culture plate format at 50,000 cells per well in fibroblast culture medium. CytoTune-iPS kits (Life Technologies) containing four Sendai virus vectors (Oct3/4, Sox2, Klf4, c-Myc) were used to infect fibroblasts at an MOI = 3 (transduction volume based on the specific titer of each lot), in fibroblast media. The day after infection, an additional 1 mL of fibroblast media was added to the culture. The next day, the media was switched to HUESM and depending on the severity of Sendai toxicity, MEFs were overlaid on some of the cultures. The medium was changed every day until colonies appeared. Colonies were manually picked and expanded on MEFs.

### Dural and scalp fibroblast gene expression profile

RNA was prepared using the RNeasy mini kit (Qiagen) per the manufacturers instructions. cRNA was amplified using the Illumina TotalPrep RNA Amplification Kit (Ambion) and run on an Illumina HT_12_v4 BeadChip Array (Ilumina), as per the manufacturer’s instructions. Analysis of microarray data was performed using Genome Studio software (Illumina).

### Immunostaining for pluripotency markers and alkaline phosphatase assay

For pluripotency staining, cultures were fixed using 4% paraformaldehyde (PFA, Santa Cruz) for 12 min at room temperature. After multiple PBS washes the cells were treated with PBS containing 0.1% Triton X-100 (Sigma) and 10% normal donkey serum (Jackson Immuno Research) for 1 hr. Cells were then treated with primary antibodies including Tra-160 (1:200, Millipore), SSEA-4 (1:500, Abcam), Tra-181 (1:200, Millipore), OCT-4 (1:500, Stemgent), Nanog (1:100, Cell Signaling), and SOX-2 (1:500, Stemgent). Alexa-conjugated anti-mouse or anti-rabbit IgG secondary antibodies were used (Invitrogen). AP staining was performed with the Vector Red Alkaline Phosphatase Substrate Kit (Vector Laboratories) per the manufacturers instructions. Nuclei were counterstained with Hoechst 33342 (Sigma).

### Nanostring nCounter assay

Total RNA was isolated from each iPSC line using the RNeasy kit (Qiagen) as per the manufacturers instructions. 100 ng of RNA for each sample was analyzed with the NanoString nCounter system (NanoString, Seattle, WA) using a pre-designed codeset. The codeset contains 25 probes for detection of retroviral and Sendai viral transgenes, pluripotency, spontaneous differentiation markers, and housekeeping genes [5]. Data was normalized to the geometric mean using nSolver Analysis Software v1.0 (NanoString) and compared with previous runs of a Sendai-positive control line, a fibroblast line (1043), and two human ESC lines (HUES42 and HUES16).

### *In vitro* pluripotency assay

Undirected embryoid bodies (EB) were formed by placing 10,000 iPSCs in multiple wells of a 96-well non-tissue culture treated V-bottom plate (Evergreen) containing HUESM plus 10 μM ROCKi (Stemgent), and underwent brief centrifugation. After 14 d of culturing EBs were transferred into a 6 well low attachment plates (Corning) and cultured for an additional 16 days. Once harvested EBs were fixed in 4% paraformaldehyde for 20 min at room temperature and processed in 15% and 30% sucrose solutions for one day each at 4°C. EBs were then embedded in O.C.T. (Sakura Finetek) and cryosectioned. The sections were blocked in PBS containing 0.1% Triton X-100 and 10% donkey serum for 1 hr at room temperature, followed by an overnight incubation at 4°C with antibodies identifying the 3 germ layers: SMA (1:500, DAKO), AFP (1:500, DAKO) TuJ1 (1:500, Covance), Sox17 (1:500, R&D Systems). Alexa-conjugated anti-mouse or anti-rabbit IgG secondary antibodies were used (Invitrogen) along with Hoechst 33342 counterstain. Sections were set with Vectashield Hard Set Mounting Media (Vector Laboratories).

### Teratoma assay

Two confluent wells of iPSC line ASC-7D-AD (p.25) were chemically disassociated using dispase (Gibco) and centrifuged for 4 minutes at 800 rpm. For each well, cells were resuspended as clumps in 100 μL of HUESM and added to 100 μl of Matrigel (BD Biosciences) on ice. A three-month-old NSG immune-compromised mouse (Jackson Laboratory) was anaesthetized with isofluorane and injected subcutaneously with a cell-suspension on each dorsal flank, and was sacrificed 75 d post injection. The teratoma was manually extracted, fixed in 4% PFA overnight and embedded in paraffin. Sections were stained with hematoxylin and eosin and histologically examined for developmental germ layers.

### Directed neuronal differentiation

MEFs were manually removed and iPSCs were brought to single cells suspension using Accutase (Life Technologies) and plated into a 48-well plate coated with polyornithine (100 μg/mL, Sigma Aldrich)/laminin (3 μg/mL, Invitrogen) at 25,000 cells per well, in mTeSR1 (Stem Cell Technologies) and 10 μM ROCKi. Cells were allowed to recover for 2 days before being switched to custom mTeSR1 (missing five growth factors, Stem Cell Technologies) containing 10 μM SB421542 (Stemgent) and 250 nM LDN193189 (Stemgent). Media was changed completely every 2 days. At Day 11, media was gradually switched with half feeds for the first two changes to neuronal differentiation medium (Neurobasal/B27 without retinoic acid/Glutamax (2 mM)/Penicillin-streptomycin (100 U/mL-0.1 mg/mL).

At days 14 and 21, wells were washed with PBS and fixed using 4% PFA for 12 min at room temperature. After three PBS washes, cells were blocked with 0.1% Triton X-100 and 10% normal donkey serum for 1 hr. Cells were treated with primary antibodies including PAX6 (1:300, Covance), Tuj1 (1:500, Covance), and Sox1 (1:400, Stemgent). Alexa-conjugated anti-mouse or anti-rabbit IgG secondary antibodies were utilized (Invitrogen).

## Results

**Figure 1:**
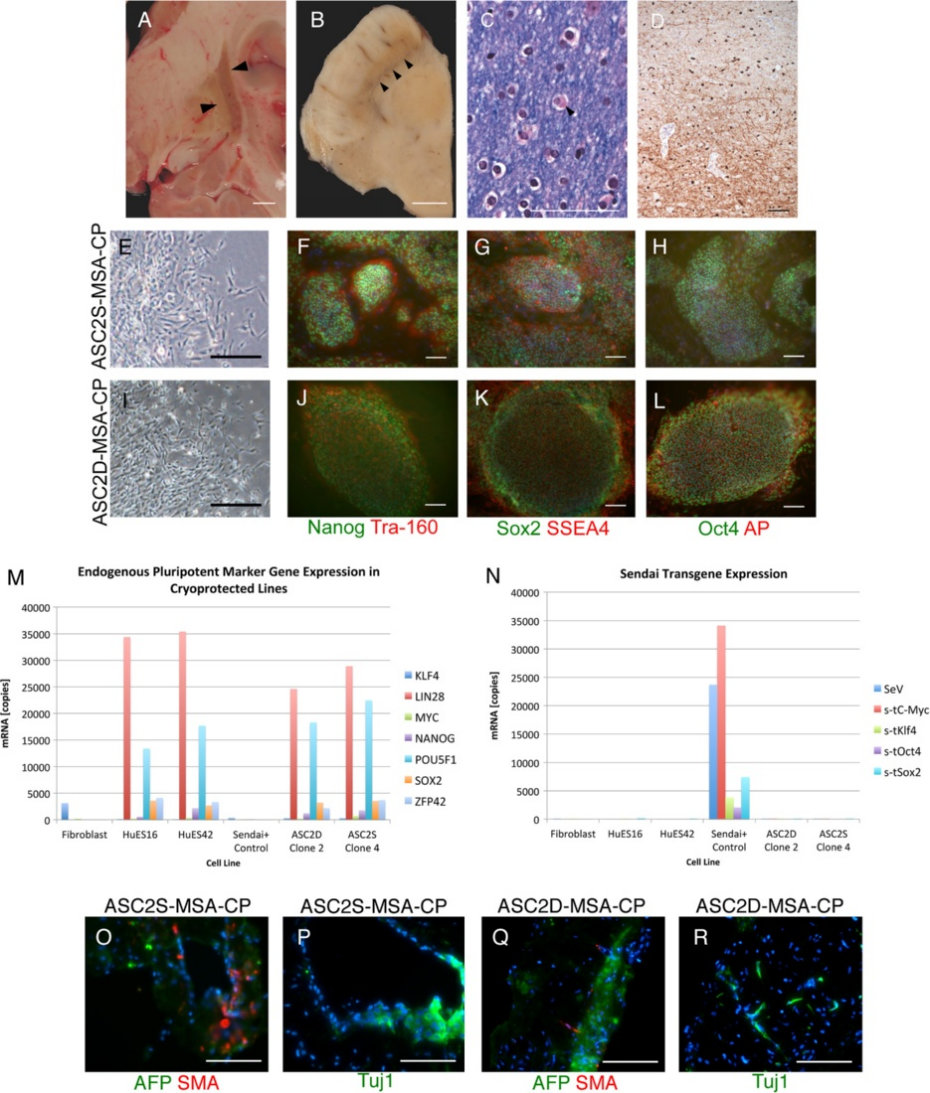
**Cryoprotected MSA patient outgrowths.** Scale bars are 1 cm for **(A-B)** and 100 μm for **(C-L, O-R)**. **(A)** Gross examination of a right coronal section through the fresh brain reveals discoloration and atrophy of the globus pallidus and putamen. **(B)** Examination of the midbrain reveals pallor of the substantia nigra. **(C)** Luxol-fast blue/hematoxylin and eosin stained sections demonstrate glial cytoplasmic inclusions (Papp-Lantos bodies) in the subcortical white matter. **(D)** These inclusions are highlighted by immunohistochemical staining to α-synuclein. **(E,I)** Outgrowths with fibroblast morphology from the scalp (ASC2S-MSA; **E**) and dura mater (ASC2D-MSA; **I**). **(F-H, J-L)** Immunostaining of scalp (ASC2S-MSA-CP) and dura (ASC2D-MSA-CP) iPSCs demonstrates expression of pluripotency markers as indicated. AP stands for alkaline-phosphatase. DNA is in blue. **(M-N)** Nanostring analysis for endogenous stem cell genes **(M)** and shutoff of Sendai transgenes **(N)**. Hues16 and Hues42 were used as positive controls for endogenous stem cell genes, unrelated fibroblasts as a negative control, and infected unrelated fibroblasts as a positive control for Sendai transgene expression. **(O-R)** Undirected EBs were cryosectioned and immunostained for the 3 developmental germ layers: endoderm (AFP), mesoderm (SMA), and ectoderm (Tuj1).

Generation of iPSCs from skin biopsies is routinely preformed by our and many other laboratories [5], but whether iPSCs with similar properties can be generated from meninges was unclear. To answer this question, we generated dural fibroblasts from the skin (scalp) and cranial dura mater tissues from a random autopsy subject from the CUMC brain bank series. This patient had multiple system atrophy (MSA), a fatal disease characterized by glial cytoplasmic inclusions composed of α-synuclein that affects the striatum and other brain regions [6] (Figure 1A-D). The tissue was frozen at autopsy using 10% DMSO/45% FBS/45% fibroblast media, a standard cryoprotection medium. Upon subsequent culture, samples taken from both tissues exhibited outgrowths with fibroblast morphology. Cell lines derived from these outgrowths were termed ASC2S-MSA and ASC2D-MSA (i.e. autopsy stem cell subject 2 skin and dura, ASC2S-MSA and ASC2D-MSA, respectively, Figure 1E,I). Gene expression profiling shows that the skin and dural fibroblast lines have similar but distinct gene expression profiles (correlation coefficient 0.86), suggesting that the endosteal-derived dural fibroblasts have unique features. Comparing nine genes commonly used as functional fibroblast markers [7] reveals that most are present at similar levels in scalp and dural cells, with the exception of FSP1 (Additional file 1). In summary, both scalp and dural tissues yield outgrowths of fibroblast identity, albeit likely of different subclasses.

Next, we attempted to reprogram skin and dural lines into a pluripotent state using a Sendai virus integration-free method [8]. After viral infection, both ASC2S-MSA and ASC2D-MSA lines produced colonies with iPS-like morphology. Individual clones were manually picked, expanded, and characterized for stem cell properties such as pluripotency (Figure 1F-H, J-R). ASC2S-MSA-CP and ASC2D-MSA-CP iPSCs (cryoprotected) also displayed a normal female karyotype and fingerprinting confirmed that both lines were derived from the same subject (Additional file 1 and data not shown). These results indicate that both scalp and dural cells can be reprogrammed to produce high-quality iPSCs.

**Figure 2:**
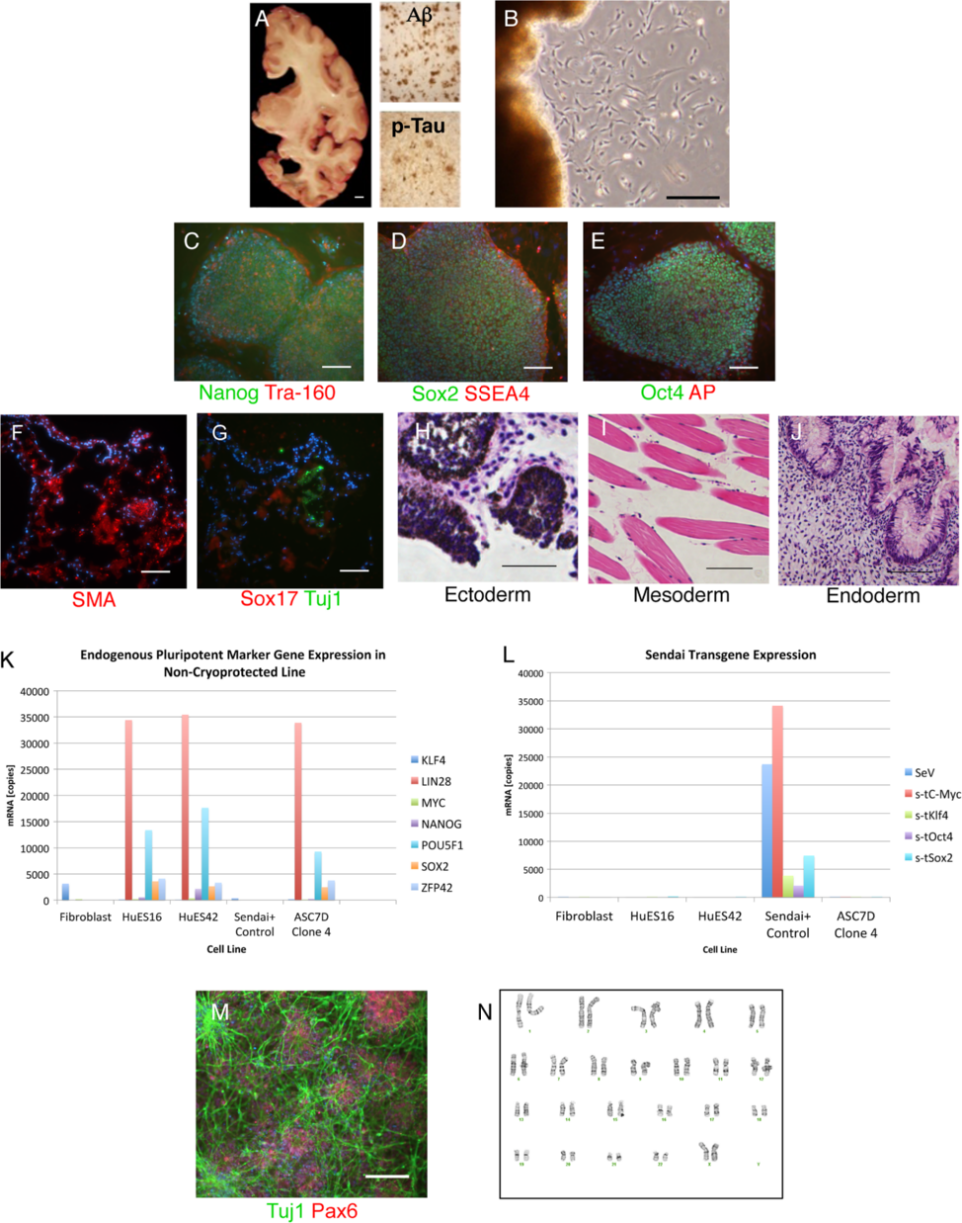
**Generation of iPSCs from non-cryoprotected dura.** Scale bars are 1 cm for **(A)** and 100 μm for **(B-M)**. **(A)** Gross examination of a coronal right hemi-section from the brain of a sporadic AD patient (case ASC7) illustrates atrophy and ventricular dilatation. Immunohistochemical staining for amyloid-β (Aβ) peptide and hyper-phosphorylated tau confirms pathological accumulation of amyloid plaques and neurofibrillary tangles (insets). Scale bar is 1 cm. **(B)** Fibroblast-like outgrowths from the thawed archived dura from the same subject, 18 days post plating. **(C-N)** Characterization of a representative iPSC clone (clone 4) derived ASC7D-AD. **(C-E)** Immunofluorescence staining using antisera targeting **(C)** Nanog (green), Tra160 (red); **(D)** Sox2 (green), SSEA4 (red); **(E)** Oct4 (green) alkaline phosphatase (red) confirms pluripotency. Nuclei are counterstained with Hoechst 33342 (blue). **(F-G)** Undirected EBs were cryosectioned and immunostained for the three developmental germ layers: endoderm (Sox17), mesoderm (SMA), and ectoderm (Tuj1). **(H-J)** Teratomas were sectioned and hematoxylin and eosin stained, and show evidence of the presence of the three developmental germ layers as indicated. **(K-L)** Nanostring analysis for endogenous stem cell genes **(K)**, and shutoff of Sendai transgenes **(L)**. **(M)** Immunofluorescence staining using antisera targeting neuron-specific class III β-tubulin (Tuj1, green) and the neural progenitor marker paired box 6 (PAX6, red) demonstrates directed neuronal differentiation (21 days). Nuclei are counterstained with DAPI (blue). **(N)** This iPSC line displays a normal female karyotype.

Based on these results, we attempted to generate iPSCs from nine additional dural samples obtained from control individuals or from subjects with late-onset AD, all of which had been frozen and archived without a cryoprotectant such as DMSO, using a commonly utilized liquid nitrogen vapor sandwich method [3,4]. We obtained successful fibroblast outgrowths from four of the nine samples, although one of these outgrowths produced insufficient cells to reprogram (ASC9D). A second line was lost to contamination (ASC8D). When the remaining two lines were infected with Sendai virus, one (ASC7D-AD) formed colonies with iPSC morphology. This sample, stored at −80°C for 9 years, was from a patient with pathological changes typical of AD (sporadic late-onset), including aggregated frequent amyloid plaques (CERAD plaque score C [9]) and severe accumulation and progression of phospho-tau inclusions (Braak neurofibrillary tangle stage VI, Figure 2A [10]). Three individual clones were manually picked and expanded with the clone displaying the best morphology selected for further characterization, including confirmation of endogenous expression of stem cell genes by immunostaining and Nanostring analysis [5], verification of Sendai transgene shutoff, establishing pluripotency both *in vitro* (embryoid body assay) and *in vivo* (teratoma), and directed differentiation into neurons (Figure 2B-M). ASC7D-AD had a normal female karyotype and matched the parent dural fibroblasts by fingerprinting (Figure 2N and data not shown).

To replicate our finding that non-cryoprotected archival dura mater can be used to generate iPSCs, we acquired 18 additional biobanked cranial dura samples from a cohort of subjects with an assortment of neuropathologies. We obtained fibroblast outgrowths from eight of these tissues (Table 1). As with our first attempt, it took longer to produce outgrowths than fresh skin biopsies: 11–30 days with an average of 17 days for frozen dural samples as compared to 5–10 days for fresh skin biopsies.

**Table 1:** Successful dural outgrowths and iPSC generation

**Sample**	**CP**	**Sex**	**Age**	**Class.**	**iPSCs**	**PMI (Frozen)**
ASC2S/D	Y	F	47	MSA	3+ clones	14’24”
ASC7D	N	F	79	AD	3+ clones	7’40”
ASC8D	N	F	54	Control	Contaminated	15’40”
ASC9D	N	F	78	AD	Insufficient #	34’55”
ASC12D	N	M	68	Control	Failed	20’46”
ASC14D	N	F	78	AD	N/A	34’55”
ASC15D	N	F	79	AD	N/A	7’40”
ASC19D	N	M	60	ALS	2+ clones	11’45”
ASC21D	N	M	89	Control	Failed	7’17”
ASC22D	N	F	54	Control	Failed	15’40”
ASC24D	N	M	72	DLBD	Failed	23’55”
ASC27D	N	M	63	HD	2+ clones	14’55”
ASC30D	N	F	76	PD	2+ clones	13’34”

***Abbreviations***. For sample, S refers to skin as tissue of origin, D refers to dura mater. CP stands for cyroprotection. Class stands for classification, which includes MSA (multiple systems atrophy), AD (Alzheimer’s disease), ALS (amyotrophic lateral sclerosis), DLBD (diffuse Lewy body disease), HD (Huntington’s disease), and PD (Parkinson’s disease). For iPSC generation, N/A (not applicable) indicates no reprogramming was attempted although the number of outgrowth cells was sufficient. PMI refers to post mortem interval, and refers to the amount of time after death before the sample was frozen in hours (’) and minutes (”).

**Figure 3:**
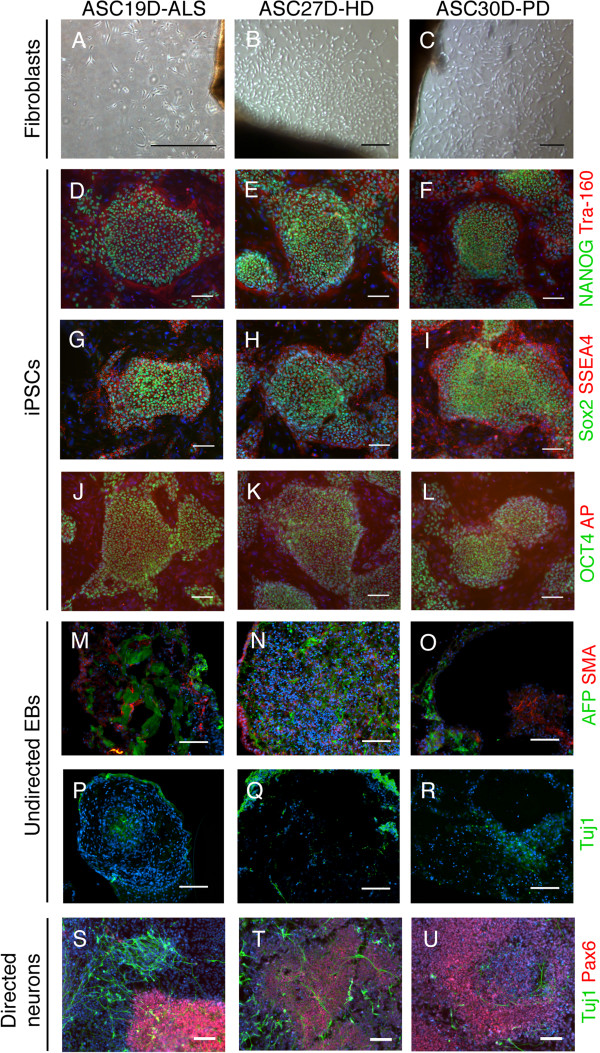
**Characterization of additional dura-derived iPSCs.** All scale bars are 100 μm and representative clones are shown. ASC19D-ALS (clone 1) is from a sporadic ALS case. ASC27D-HD (clone 2) is from a Huntington’s disease case. ASC30D-PD (clone 1) is from a sporadic Parkinson’s disease case. **(A-C)** Outgrowth with fibroblast morphology from dura mater. **(D-L)** Immunostaining for pluripotency markers as indicated. AP stands for alkaline-phosphatase. DNA is in blue. **(M-R)** Undirected EBs were cryosectioned and immunostained for the three developmental germ layers: endoderm (AFP), mesoderm (SMA), and ectoderm (Tuj1). **(S-U)** iPSC-derived neurons (Tuj1) and neural progenitors (Pax6) after 14 days of directed neuronal differentiation. DNA is in blue.

Sendai virus-mediated reprogramming was successful in three of six lines, including samples from neuropathologically-confirmed sporadic amyotrophic lateral sclerosis (ALS), Huntington’s disease, and Parkinson’s disease (Table 1). These tissues had been stored at −80°C for 10–11 years. Post mortem interval before freezing ranged between 4.5 hours and 39 hours, and did not correlate with either successful outgrowth or subsequent reprogramming. Two clones from each line were manually picked and expanded, and the clone with best morphology was further characterized. Similar to what was done for ASC2D/S-MSA and ASC7D-AD reprogramming, additional clones (at least 2+ per line) were frozen in bulk for potential future use. While the reprogramming efficiency was not as high as fast growing fresh skin biopsies (easily 20+ colonies under good conditions), it was sufficient to allow one to study a handful of clones if desired. In each case, Nanostring assays and immunostaining for endogenous stem cell markers confirmed successful reprogramming and Sendai transgene shutoff, embryoid bodies confirmed pluripotency, and iPSCs had the capacity to undergo directed differentiation into neural cells (Figure 3 and Additional file 2). ASC19D-ALS (amyotrophic lateral sclerosis) and ASC30D-PD (Parkinson’s disease) cell lines had normal karyotypes, although multiple clones of ASC27D-HD (male Huntington’s disease subject) lacked the presence of a Y chromosome (data not shown). PCR amplification of the *AMG* gene, which can produce different amplicon sizes depending if *AMG* is located on the X or Y chromosome, suggests that the Y chromosome was lost in the fibroblast outgrowth culture, but is present in genomic DNA from the original dural tissue (data not shown). In summary, fourteen of twenty-five (56%) dural samples produced fibroblast outgrowths, and four of nine (44%) outgrowths were successfully reprogrammed.

## Discussion

These results demonstrate the potential to leverage existing biorepositories to support research in a powerful new way. While tissue banks in various forms have existed for over 60 years, demand for human tissue for personalized medicine and associated genetic studies, which require large biorepositories, has accelerated the rate of establishment of new facilities [11,12]. A recent survey has identified at least 636 U.S. biobanks, 36% of which have postmortem tissue, 9% exclusively (largely brain banks) [11]. Archival specimens that were not intended for iPSC generation at the time of harvesting have the potential to be unlocked for functional studies to test mechanistic hypotheses. Preliminary results suggest other banked brain tissues, including leptomeninges and the large artery associated with the choroid plexus, can also produce outgrowths both morphologically similar and distinct from fibroblasts (data not shown). In addition, while many collections store solid tissues, there are other specimens within these banks that might be suitable for generating stem cell lines in the future, including blood, saliva or buccal cells, cord blood and pathological body fluids (e.g, ascites), among others [11]. The majority of biobanks focus on a single disease group, with neoplasia, neurodegeneration and HIV/AIDS being the most common, but there is a rich diversity of diseases represented in these collections that include both common and rare diseases, many of which currently have no cellular models. In the case of Alzheimer’s disease, the Alzheimer’s Disease Education and Referral Center (ADEAR) currently lists 27 NIA funded Alzheimer’s disease centers, each containing a neuropathology core that routinely banks frozen tissue (http://www.nia.nih.gov/research/dn/alzheimers-disease-centers-adcs). While the total number of specimens available for generating iPSCs is difficult to estimate, the National Alzheimer’s Coordinating Center (NACC) has autopsy data from ~13,279 subjects from these centers as of June 1^st^, 2013 (https://www.alz.washington.edu/WEB/data-descript.html), the majority of which have frozen tissue available.

## Conclusions

There is a vital need for well-characterized patient material for translational research [13]. Deriving iPSCs from tissue from patients with neurodegenerative diseases with post-mortem confirmation, which remains the gold standard, is highly advantageous over utilization of lines from patients with clinical ascertainment alone in that there is certainty in the diagnosis. This approach has the additional benefit of having post-mortem brain tissue available for rapid correlation and cross validation of neuropathological and cellular findings.

## Competing interests

The authors declare they have no competing interests.

## Author contributions

AS conceived and performed experiments, analyzed data, oversaw research, and drafted/collated the final manuscript. LV conceived and performed experiments, analyzed data, and helped draft and edit the manuscript. CD and DP performed experiments and contributed to experimental design, analyzed data, and helped draft and edit the manuscript. SJ performed teratoma experiments and contributed to experimental design, and prepared data panels for publication. JV provided pathological images, provided samples via the NYBB, and helped edit the manuscript. MS provided intellectual input into the manuscript, helped edit the manuscript, and provided funding. JC conceived the experimental approach, coordinated the cryoprotected pilot, provided pathological images and helped draft and edit the manuscript. SN provided intellectual input into the manuscript, oversaw research, helped edit the manuscript, and provided funding. All authors read and approved the final manuscript.

## Supplementary Material

Additional file 1 **Related to Figure **1**.** Characterization of scalp and dura-derived iPSCs. This figure shows the relative expression of fibroblast genes for scalp and dural outgrowths from the same MSA patient, as shown in Figure 1, as well as karyotype data for these lines. Click here for file

Additional file 2 **Related to Figure **3**.** Additional characterization of dura-derived iPSCs. This figure shows additional Nanostring analysis for endogenous stem cell genes and shutoff off Sendai transgenes for additional iPSC lines. Click here for file
