# Supplementary material for: Generation of iPSC lines from archived non-cryoprotected biobanked dura mater
Source: Acta Neuropathol Commun. 2014 Jan 7;2:4. doi: 10.1186/2051-5960-2-4 (PMC3895779; doi:10.1186/2051-5960-2-4)
Supplement: Additional file 1 — Related to Figure 1. Characterization of scalp and dura-derived iPSCs. This figure shows the relative expression of fibroblast genes for scalp and dural outgrowths from the same MSA patient, as shown in Figure 1, as well as karyotype data for these lines. [file 2051-5960-2-4-S1.pdf]

A

Gene	Scalp	Dura
S100A4 (FSP1)	1	0.07
ACTA2	1	0.44
ANPEP (CD13)	1	0.56
COL1A1	1	1.21
COL1A2	1	1.16
Desmin	1	0.55
DDR2	1	0.92
ITGA1	1	0.64
ITGB1	1	0.84

B

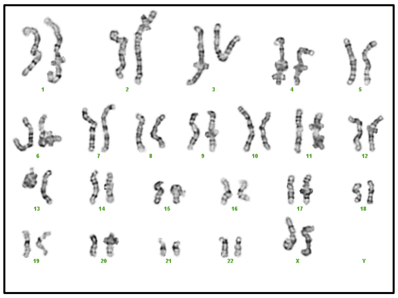

ASC2S-MSA-CP
(iPSC)

C

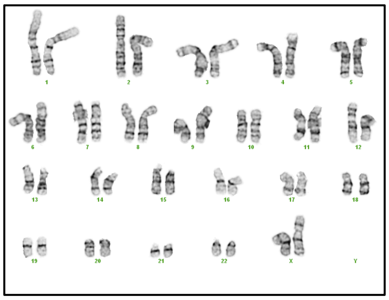

ASC2D-MSA-CP
(iPSC)

Additional File 1, Related to Figure 1: Characterization of Scalp and Dura-Derived iPSCs. (A) RNA isolated from scalp and dural fibroblast lines from from the same patient (ASC2S and ASC2D) were analyzed on the Illumina HumanHT-12-14 BeadChip platform as biological triplicate samples. Scorecard analysis comparing scalp and dural fibroblast microarray expression data for representative fibroblast markers. (B-C) ASC2D-MSA-CP (clone 2) and ASC2S-MSA-CP (clone 3) both display a normal female karyotype.
