# Supplementary material for: Generation of iPSC lines from archived non-cryoprotected biobanked dura mater
Source: Acta Neuropathol Commun. 2014 Jan 7;2:4. doi: 10.1186/2051-5960-2-4 (PMC3895779; doi:10.1186/2051-5960-2-4)
Supplement: Additional file 2 — Related to Figure 3. Additional characterization of dura-derived iPSCs. This figure shows additional Nanostring analysis for endogenous stem cell genes and shutoff off Sendai transgenes for additional iPSC lines. [file 2051-5960-2-4-S2.pdf]

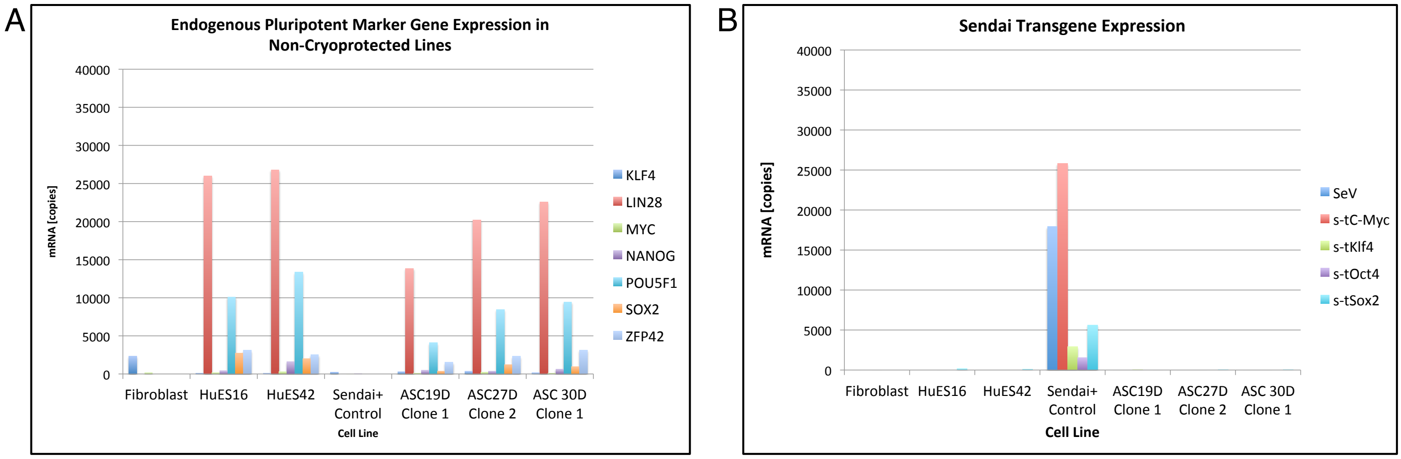

Additional File 2, Related to Figure 3: Additional characterization of dura-derived iPSCs. (A) Nanostring analysis for endogenous stem cell genes and (B) shutoff of Sendai transgenes for ASC19D-ALS (clone 1), ASC27D-HD (clone 2), and ASC30D-PD (clone 1). Hues16 and Hues42 were used as positive controls for endogenous stem cell genes, unrelated fibroblasts as a negative control, and infected unrelated fibroblasts as a positive control for Sendai transgene expression.
